# Crude incidence in two-phase designs in the presence of competing risks

**DOI:** 10.1186/s12874-015-0103-1

**Published:** 2016-01-11

**Authors:** Paola Rebora, Laura Antolini, David V. Glidden, Maria Grazia Valsecchi

**Affiliations:** Center of Biostatistics for Clinical Epidemiology, School of Medicine and Surgery, University of Milano-Bicocca, via Cadore 48, Monza, 20900 Italy; Department of Epidemiology and Biostatistics, University of California, San Francisco, California USA

**Keywords:** Two-phase design, Competing risks, Crude incidence, Case-control, Case-cohort, Missing data, Subdistribution hazard

## Abstract

**Background:**

In many studies, some information might not be available for the whole cohort, some covariates, or even the outcome, might be ascertained in selected subsamples. These studies are part of a broad category termed two-phase studies. Common examples include the nested case-control and the case-cohort designs. For two-phase studies, appropriate weighted survival estimates have been derived; however, no estimator of cumulative incidence accounting for competing events has been proposed. This is relevant in the presence of multiple types of events, where estimation of event type specific quantities are needed for evaluating outcome.

**Methods:**

We develop a non parametric estimator of the cumulative incidence function of events accounting for possible competing events. It handles a general sampling design by weights derived from the sampling probabilities. The variance is derived from the influence function of the subdistribution hazard.

**Results:**

The proposed method shows good performance in simulations. It is applied to estimate the crude incidence of relapse in childhood acute lymphoblastic leukemia in groups defined by a genotype not available for everyone in a cohort of nearly 2000 patients, where death due to toxicity acted as a competing event. In a second example the aim was to estimate engagement in care of a cohort of HIV patients in resource limited setting, where for some patients the outcome itself was missing due to lost to follow-up. A sampling based approach was used to identify outcome in a subsample of lost patients and to obtain a valid estimate of connection to care.

**Conclusions:**

A valid estimator for cumulative incidence of events accounting for competing risks under a general sampling design from an infinite target population is derived.

## Background

In many longitudinal studies, some information might not be measured/available for the whole cohort, in fact biomarkers/additional covariates, or even outcome, might be ascertained only in selected subsamples. These studies are part of a broad category termed two-phase studies [1], in fact they imply two sampling phases: the first one being usually a random sample from the target population, ending up in the entire cohort (phase I sample), and the second one applying some kind of sampling (e.g. efficient or of convenience) to collect additional information or the selection of subjects with no missing data (phase II sample). Common examples of efficient second phase sampling include the nested case-control and the case-cohort designs [2–5]. In other situations the outcome itself is collected only for a subsample [6]. Two-phase sampling, or more generally, multiphase sampling, is a general design that includes any valid probability sample of the data, in which each subsampling can depend on all the currently observed data at each step [7]. The actual sampling probabilities will depend on the specific design. The acknowledgement of these sampling phases, even in the commonly applied designs, can be very useful to improve efficiency and to allow flexibility in the analysis (e.g. different time-scales or different models can be applied) by using information available for the whole cohort [8].

Efficient designs are particularly useful to identify new biomarkers when the combination between large cohorts and expensive new technologies make it infeasible to measure the biomarkers on the entire cohort. The Women’s Health Initiative program, for example, stored serum and plasma from participants and used them for specialized studies [9]. Also the Cardiovascular Health Study collected DNA from most participants to study different genetic factors underlying cardiovascular or other diseases and only subsets of the cohort have been genotyped in different projects [10].

In our first motivating clinical example, the aim was to evaluate the role of different genetic polymorphisms on treatment failure due to relapse in childhood acute lymphoblastic leukemia (ALL) using clinical information and biological samples available from a clinical trial that enrolled nearly 2000 patients. In this situation, a parsimonious use of these specimens motivated the choice of an efficient/optimal two-phase sampling design [11]. We present also a further application where the aim was to estimate engagement to care of HIV patients in resource limited settings. Here the outcome itself was missing in a group of patients due to possibly informative loss to follow-up. The outcome was tracked in a random sample of those lost to follow-up to obtain a valid estimate of engagement to care [12].

For two-phase studies, appropriate weighted survival estimates have been derived, both in the presence of additional covariates measured in the second phase [7, 13, 14], as well as in cohorts where the outcome/follow-up is not available for everyone [15]. A Cox model adapted for two-phase designs has also been derived [14]. However no estimator of cumulative incidence accounting for competing events in the general framework of two-phase designs has been proposed, while it has been developed for specific designs, such as nested case-control studies [16, 17]. This is relevant in the presence of multiple types of events, such as relapse and (toxic) death in cancer patients, as in the motivating examples presented here, where estimation of event type specific quantities are needed for evaluating outcome.

The aim of this paper is to develop a non parametric estimator of the crude incidence of events accounting for possible competing events in the general framework of two-phase designs, where subgroups of analysis might be defined according to explanatory variables ascertained in the phase II sample, or the outcome itself assessed only in the second phase sample.

In the Methods section we propose a weighted crude incidence estimator for application in two-phase designs. The theoretical properties of the proposed method are derived in appendix and investigated through simulations under different scenarios, which results are reported in Results section. In this section we also report the examples on childhood ALL and HIV patients. Conclusions is dedicated to the discussion.

## Methods

### Notation and basics

Let *T* be the failure time variable and suppose there are *K* possible causes of failure denoted by *ε*=1,2,…*K*. Let the cause-specific hazard function of the *k*^*th*^ event be:

$$\lambda_{k}(t)={\lim}_{dt \rightarrow 0} \frac{1}{dt}P(t\leq T< t+dt;\varepsilon=k|T\geq t) $$

and $\Lambda _{k}(t)={\int _{0}^{t}} \lambda _{k}(s)ds$. Define

(1) $$ F_{k}(t)=P(T\leq t;\varepsilon =k)   $$

as the probability that a failure due to cause *k* occurs by time *t*, that is the quantity that we aim to estimate. Define also $S(t)=P(T>t)=1-\sum _{k} F_{k}(t)$ as the probability of surviving from any cause of failure.

A convenient representation of the crude incidence function (1) as product limit estimator naturally arises starting from the subdistribution hazard introduced by Gray [18] and defined as:

(2) $$ \begin{aligned}  \lambda_{k}^{*}(t)&={\lim}_{dt \rightarrow 0} \frac{1}{dt}P\{t\leq T< t+dt;\varepsilon\\ &=k|T\geq t\cup (T < t;\varepsilon \neq k)\} \end{aligned}  $$

This hazard has been shown to be very useful to compare the crude cumulative hazard functions in different groups, since it restores a one-to-one relationship between the hazard and the cumulative probability of a particular failure type: , with $\Lambda _{k}^{*}(t)={\int _{0}^{t}} \lambda _{k}^{*}(s)ds$ and where the product integral notation  is used to suggest a limit of finite products $\prod $ [18, 19]. Of note, the one-to-one relationship between the hazard and the cumulative probability is not satisfied from the cause-specific hazard in the presence of competing events [20]. The subdistribution hazard can be thought as the hazard of an artificial variable $T^{*}_{k}=T\cdot I\left \{ \varepsilon =k\right \} +\infty \cdot I\left \{ \varepsilon \neq k \right \}$ that extends to infinity the time to event *k* when another competing event is observed. In fact, for any finite *t*, $T^{*}_{k}\leq t$ is equivalent to *T*≤*t* and *ε*=*k*; thus, given definition (1), $P\left (T^{*}_{k}\leq t\right) = F_{k}(t)$. The definition of $T^{*}_{k}$ is consistent with the argument that when an event other than *k* occurs as first, the latter will never be observed as first and thus the corresponding time is infinity.

Let (*T*_*i*_,*ε*_*i*_,*C*_*i*_,*Z*_*i*_), with *i*=1…*N*, be *N* independent replicates of (*T*,*ε*,*C,Z*), where *C* is the censoring time and *Z* a vector of covariates. We will refer to these *N* subjects as the phase I sample. Define *X*= min(*T,C*) and *Δ*=*I*(*T*≤*C*). We will assume that failure and censoring times are conditionally independent, *T*⊥*C*|*Z*. Let *Y*_*i*_(*t*)=*I*(*X*_*i*_≥*t*), *N*_*ik*_(*t*)=*I*(*X*_*i*_≤*t*,*Δ*_*i*_*ε*_*i*_=*k*) and $N_{i \cdot }(t)=\sum _{k=1}^{K} N_{\textit {ik}}(t)$, where *I*(·) is the indicator function. Define *G*(*t*)=*P*(*C*>*t*) as the probability to remaining uncensored up to *t*.

Suppose that complete information on (*X*_*i*_,*Δ*_*i*_*ε*_*i*_,*Z*_*i*_) is available only for a subset *n*<*N* of subjects drawn based on a possibly complex sampling design and let *ξ*_*i*_ indicate whether subject *i* is selected into this sample. We will refer to the $n=\sum _{i}\xi _{i}$ subjects as the phase II sample, even if multiple phases of sampling could actually be involved to obtain the final complete sample [7]. Let *π*_*i*_=*P*(*ξ*_*i*_=1|*X*_*i*_,*Δ*_*i*_*ε*_*i*_,*Z*_*i*_) being the inclusion probability of subject *i* for the phase II sample, conditional on being selected at the first phase. In a random sample this probability is equal for every subject. However sampling is often stratified on some variables to increase efficiency; in this case, the probability to be selected for the phase II sample is common for all subjects in the same stratum and differs between strata. In particular, it is usually higher for the more informative strata (e.g. strata including subjects with the event of interest as in case-control studies). For nested case-control designs the sampling probability of cases will be 1, while the one of controls might be derived as the probability that individual *i* is ever selected as control, following Samuelsen [4]. We denote the pairwise sampling probability for any two subjects (*i,j*, with *i*≠*j*) by *π*_*ij*_=*P*(*ξ*_*i*_=1,*ξ*_*j*_=1|*X*_*i*_,*Δ*_*i*_*ε*_*i*_,*Z*_*i*_,*X*_*j*_,*Δ*_*j*_*ε*_*j*_,*Z*_*j*_). As commonly assumed in survey theory, the sampling method should have the following properties: the sampling probabilities *π*_*i*_ and *π*_*ij*_ must be non zero for all *i,j* in the population and must be known for each *i,j* in the sample [7].

### Incidence estimation in the presence of competing risks

#### Overall survival/incidence estimate

Under a two-phase design it is common to be interested in estimating survival in subgroups related to variables ascertained only in phase II sample (i.e. biomarkers). Another possible situation is that, instead of covariates, the outcome itself is not available for the whole cohort. Thus, in both cases an estimate of the incidence of event using only the phase II sample is very useful. The total number of events of type *k* up to *t* and the total number of persons at risk at time *t* for the entire phase I sample can be estimated from the phase II sample (accounting for the sample design) by $\hat {N}_{\cdot k}(t)=\sum _{i=1}^{N} [\!\xi _{i} N_{\textit {ik}}(t)/\pi _{i}]$ and $\hat {Y}_{\cdot }(t)=\sum _{i=1}^{N} [\!\xi _{i} Y_{i}(t)/\pi _{i}]$, respectively. Note that these estimates are valid under general sampling designs, where *π*_*i*_ and *π*_*ij*_, the so-called ‘design weights’, are known for the observations actually sampled [21].

The estimate of the overall survival has been shown by several authors in different contexts of complex sampling [13–15]: 
(3)

where the overall hazard can be obtained by $\hat {\Lambda }(t)=\sum _{k=1}^{K} \hat {\Lambda }_{k}(t)$ and $\hat {\Lambda }_{k}(t)= {\int _{0}^{t}} \hat {N}_{\cdot k}(ds)/\hat {Y}_{\cdot }(s)$ [22]. It has been shown that $\sqrt {N}[\!\hat {\Lambda }(t)-\Lambda (t)]$ converges weakly to a zero-mean Gaussian process [14, 15].

#### Competing risk

The goal is to estimate the crude incidence of a given cause *k*, , using the phase II sample, which is also called subdistribution function and is the probability that a failure due to cause *k* occurs within *t* [23, 24]. The estimate of ${\Lambda }_{k}^{*}(t)$ is based on the count of events due to cause *k* and the count of subjects at risk for $T^{*}_{k}$, denoted by $\hat {Y}_{\cdot k}^{*}(s)$ (see Appendix A.1):

(4) $$\hat{Y}^{*}_{\cdot k}(s)= \sum_{i=1}^{N} \frac{\xi_{i}}{\pi_{i}} Y_{i}(s) + \sum_{i=1}^{N} \frac{\xi_{i}}{\pi_{i}} \left[ \sum_{l\neq k} N_{il}(s^{-})\cdot \hat{G}(s^{-}|X_{i}^{-})\right]  $$

The estimate of the cumulative subdistribution hazard in (2) can now be estimated, using only the phase II sample, by: 
(5)

Note the complement of *F*_*k*_(*t*) can be thought as the survival probability of $T^{*}_{k}$ [18, 20, 25], thus a product limit type estimator can be directly derived as: 
(6)

Interestingly, this estimator is algebraically equivalent to the Aalen-Johansen type estimator, shown by [18] for random sampling, and in the Appendix A.2 for general sampling: 
(7)

It is easy to see that in the absence of competing events, $\hat {Y}^{*}_{\cdot k}(s)$ in (4) degenerates to the usual risk set $\hat {Y}_{\cdot }(s)$, thus $\hat {\Lambda }_{k}^{*}(t)=\hat {\Lambda }(t)$ and $\hat {F}_{k}(t)$ equals the complement of 1 of the weighted Kaplan-Meier estimator for two-phase studies [13]. Under no censoring, the weight $\hat {G}(s^{-}|X_{i})$ becomes 1 and the risk set $Y^{*}_{\cdot k}$ is eroded in time only by events of type *k*, therefore $\hat {F}_{k}(t)$ degenerates into the proportion of events of type *k* estimated by the phase II sample (weighted number of events of type *k* out of the estimated total size of the cohort, phase I). If every subject in phase I is sampled (*ξ*_*i*_=1 ∀*i*), then (5) becomes the standard subdistribution cumulative hazard [19, 20] and (6) the standard estimator of the crude incidence.

For simplicity of notation, in (6) we estimated the overall incidence regardless of covariates, but the estimator can also be applied on subgroups defined by *Z*. The censoring probability *G*(*t*) should also be estimated in subgroups defined by *Z*. The overall estimator is reasonable when we make the more restrictive assumption *T*⊥*C*, otherwise separate estimators conditional on *Z* would be more appropriate (and eventually an average, weighted on the frequencies of Z, between the conditional estimates).

#### Variance and confidence intervals

Following Breslow and Wellner [26], we can express $\sqrt {N}\left [\hat {\Lambda }^{*}_{k}(t)-\Lambda ^{*}_{k}(t)\right ]=\sqrt {N}\left [\tilde {\Lambda }^{*}_{k}(t)-\Lambda ^{*}_{k}(t)\right ]+ \sqrt {N}\left [\hat {\Lambda }^{*}_{k}(t)-\tilde {\Lambda }^{*}_{k}(t)\right ]$ where $\tilde {\Lambda }^{*}_{k}(t)$ represents the crude cumulative incidence estimator that we would have obtained if complete information (*X*_*i*_,*Δ*_*i*_*ε*_*i*_,*Z*_*i*_) was known for all the subjects in phase I sample (*i*=1…*N*) [18]. The two terms are asymptotically independent [14, 26]. The first term converges weakly to a zero-mean Gaussian process [19] with covariance that we denote as $\sigma _{k\medskip I}^{2}(t)$. By the arguments in Appendix A.3, the second term converges weakly to a zero-mean Gaussian process with covariance $\sigma _{k\medskip II}^{2}(t)$. Hence, $\sqrt {N}\left [\hat {\Lambda }^{*}_{k}(t)-\Lambda ^{*}_{k}(t)\right ]$ converges weakly to a zero-mean Gaussian process with covariance being the sum of the contribution of each sampling phase: ${\sigma _{k}^{2}}(t)=\sigma _{k\medskip I}^{2}(t)+\sigma _{k\medskip II}^{2}(t)$. The first one represents the irreducible minimum uncertainty that would remain if everyone in phase I would be sampled and the second one accounts for the fact that complete information is available only in the phase II sample [13, 14, 26].

Each contribution to the variance can be estimated by the influence function approach [27]. The influence function of an estimator describes how the estimator changes when single observations are added or removed from the data and has the property that the difference between the estimate and the population quantity can be expressed as the sum of influence functions over all the subjects in the sample. By denoting with $z_{\textit {ik}}^{*}(t)$ the influence function of subject *i* on $\hat {\Lambda }^{*}_{k}(t)$ we have that [28]:

(8) $$ \hat{\Lambda}_{k}^{*}(t)-\Lambda^{*}_{k}(t)=\sum_{i=1}^{N} z_{ik}^{*}(t)+o(1/\sqrt{N})  $$

The influence function of subject *i* on $\hat {\Lambda }^{*}_{k}(t)$ has been derived in Appendix A.4.

By using the Horvitz-Thomposon variance [29] on the weighted influence function, the contribution of the variance of phase II will be:

(9) $$ \begin{aligned} \hat{\sigma}_{k\medskip II}^{2}(t) &=\hat{var}\left[\sum_{i=1}^{N} \frac{\xi_{i}}{\pi_{i}} z_{ik}^{*}(t)\right]=\\ &=\overset{n}{\underset{i=1}{\sum}} \sum_{j=1}^{n} \left[\frac{z_{ik}^{*}(t)\cdot z_{jk}^{*}(t)}{\pi_{i}\cdot \pi_{j}} - \frac{z_{ik}^{*}(t)\cdot z_{jk}^{*}(t)}{\pi_{ij}}\right]  \end{aligned}  $$

For phase I, the variance $\hat {\sigma }_{k\medskip I}^{2}(t) $ can also be estimated using (9) by setting sampling probabilities to 1 [13].

Given the one-to-one relationship between *F*_*k*_(*t*) and $\Lambda _{k}^{*}(t)$, the variance of the crude cumulative incidence (6) can now be estimated as:

(10) $$ \hat{var}\left[\hat{F}_{k}(t)\right]=\left[1-\hat{F}_{k}(t)\right]^{2}\cdot\hat{\sigma}_{k}^{2}(t)  $$

In analogy with the survival estimate for two-phase designs, we derived confidence intervals for (6) on the logarithm scale by:

(11) $$ \exp \left\{\log[\hat{F}_{k}(t)] \pm q_{\alpha/2}\frac{1-\hat{F}_{k}(t)}{\hat{F}_{k}(t)} \hat{\sigma}_{k}(t)\right\}  $$

where *q*_*α*/2_ denotes the *α*/2 quantile of the standard Gaussian distribution.

### Software

By using suitable weights for both study design and censoring, any software allowing for time dependent weights can be used to derive the modified risk set and to estimate the crude cumulative incidence function (6). These weights have been implemented in R in function crprep in the mstate package [30] and in STATA in the stcrprep function. However, any software can be used to derive the ingredients for the modified risk set in (4) and these can be used to estimate (6) and its variance by the Horvitz-Thompson approach.

The complete code to compute this estimate has been developed in R software [31] using the survey package [32] and is available at [33]. An example of the application of this function is given in the subsection Genotype ascertained on a subset of a clinical trial cohort.

## Results

### Simulations

#### Simulations protocol

We considered two competing events with independent latent times *T*_1_ and *T*_2_ and constant marginal hazard of 0.1, the crude incidences are then $F_{1}(t)=F_{2}(t)=\frac {1}{2}(1-e^{-0.1t})$. We focused on the crude incidence of event 1 up to *t*=2 units of time (i.e. years). This implies a fraction of 82 % with no events at *t*=2 (administrative censoring) and a crude incidence of about 9 %. The independence between the latent times *T*_1_ and *T*_2_ is not restrictive given the non identifiability issue [34]. The censoring time followed an uniform distribution on ranges (0.5,30.5) and (0.5,10.5), leading to around 5 % and 15 % censored before *t*=2, respectively.

We drew *B*=1000 random first-phase samples of size *N*=1000, from which we sampled a phase II sample according to different study designs:

1. random sample, with *n*=50,100 units;
2. case-control sampling: we randomly sampled *n*/2 individuals among those who experienced event 1 (cases) up to time 2 and *n*/2 individuals among the others (controls), with *n*=50,100 units;
3. stratified sampling: we considered the phase I sample divided into 4 strata defined by the variable *Z*={0,1} (with frequencies 70 *%* and 30 *%* for *Z*=0 and *Z*=1, respectively) and the occurrence or not of event 1 up to time 2. The hazard rates were assumed to be 0.08 and 0.2 with *Z*=0 and with *Z*=1, respectively. An equal number of subjects (*n*/4) were sampled for each strata (balanced sampling), with *n*=50,100 units.
4. nested case-control design: we selected all cases and *m* controls for each case with no events at the time of event of the case, fixing *m*=1,2. Under this design we cannot fix a total sample size a priori, but we expect around 90 events and 90·*m* controls. Sampling probabilities for each included subject were derived according to Samuelsen [4].

B = 1000 was chosen in order to get a ±5 % level of accuracy in the estimate of the crude incidence (*F*_1_(*t*),*t*>0.3) in about 95 % of the samples. For each sample, $\hat {F}_{1}(t)$ has been computed by (6), with $\hat {\Lambda }_{1}^{*}(dt)$ estimated by (5), and it has been compared with *F*_1_(*t*) in order to assess bias in each sample: $\hat {F}_{1b}(t)-F_{1}(t)$, *b*=1…*B*. Bias has been computed and reported for 20 different time points *t*=0.1,0.2,…,2. For each simulation, we also computed standard error of $\hat {F}_{1}(t)$ according to (10) and the 95 % confidence interval (CI) of $\hat {F}_{1}(t)$ on the logarithm scale (11) to evaluate coverage and length.

#### Simulations results

**Fig. 1:**
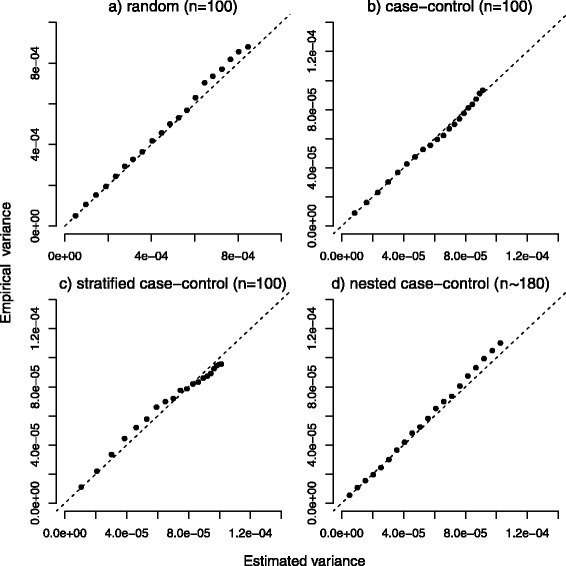
Estimated and empirical variances. Comparison between estimated and empirical variance under random (panel **a**), case-control (panel **b**), stratified (panel **c**) and nested case-control sampling (*m*=1, panel **d**). For the first three scenarios a sample size of 100 was used, while in the nested case-control sampling a mean of 180 subjects was considered. Data were subject to a random censoring of 15 % (plus administrative censoring). Dashed lines represent the main bisector corresponding to the equality between estimated and empirical reference

Figure 1 compares the average of the estimated standard error of $\hat {F}_{1}(t)$ in each simulation with the empirical standard error at the 20 different times of observation in the four different scenarios with random censoring of 15 *%*. They were found to be very close in all scenarios, as expected.

**Fig. 2:**
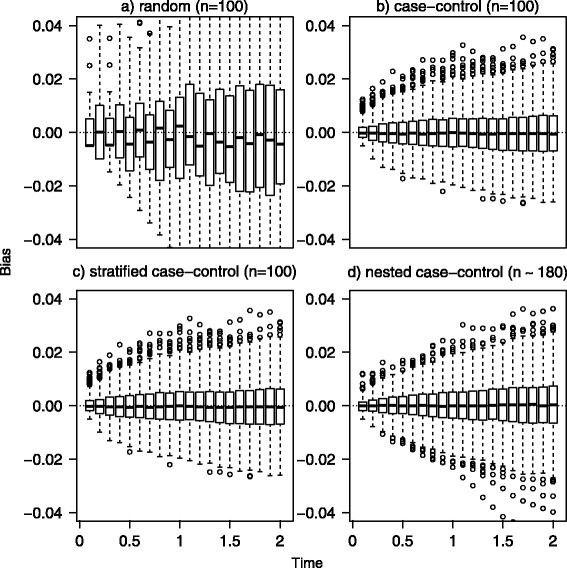
Bias distribution. Distribution of bias in the 1000 simulated samples under random (panel **a**), case-control (panel **b**), stratified (panel **c**) and nested case control sampling (*m*=1, panel **d**). For the first three scenarios a sample size of 100 was used, while in the nested case-control sampling a mean of 180 subjects was considered. Data were subject to a random censoring of 15 % (over administrative censoring). The box represent the first and third quartile, the black line the median and the empty dots represent outliers defined as bias more than 1.5 times the interquartile range above the third quartile (or more than 1.5 times the interquartile range below the first quartile). Dotted lines represent the reference for bias (bias equal to 0)

Figure 2 reports the distribution of bias in each one of the 1000 simulated samples under random (panel a), case-control (panel b), stratified (panel c) and nested case-control sampling (*m*=1, panel d). Bias fluctuates around 0 in each scenario, but it has more variability in the random sampling compared to other scenarios, resulting also in a higher mean value of absolute bias over the B simulations (still always lower than 0.2 %). The lower performance of the estimator in the first scenario is due to the fact that random sampling is not a convenient design in the simulated setting. In fact, in phase I cohort we expect around 90 events of type 1 (incidence of 9 % and sample size of 1000), thus if we randomly sample 100 subjects from the phase I cohort, we expect to observe only 9 events (in phase II sample). With such a small number of events, unbiasedness is in fact not sufficient to ensure a reasonable behaviour and to get enough information on event incidence. To address this issue, the other study designs (scenarios 2, 3, and 4) are indeed thought to guarantee to sample more events of type 1. Thus we recommend adopting efficient designs accounting for the event of interest. Relative and standardized biases were always lower than 6 % (data not shown). The mean square error, not shown, slightly increases with time, in fact variability is increasing in time (as confirmed by the empirical standard error of $\hat {F}_{1}(t)$ and by Fig. 2). The average length of the confidence interval was consistently increasing with time, ranging between 7 % and 12 % in the random sampling and between 2 % and 4 % in the case-control, stratified and nested case-control sampling (data not shown). This comparison underscores the advantages of a careful selection of the subsample.

**Fig. 3:**
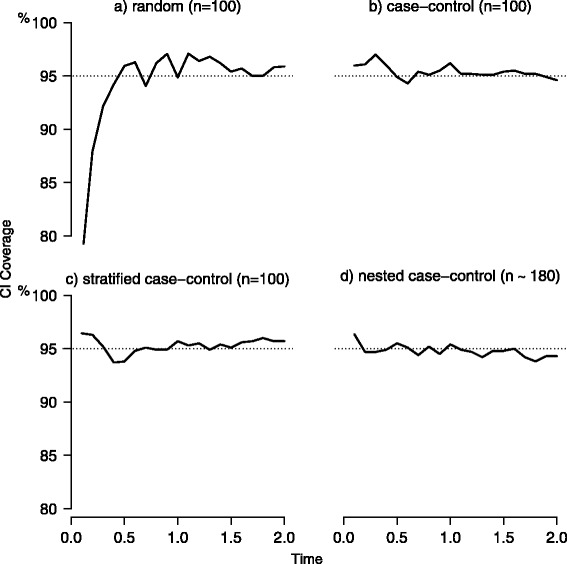
Coverage of confidence intervals. Simulation results for the coverage of confidence intervals (CI) under random (panel **a**), case-control (panel **b**), stratified (panel **c**) and nested case-control sampling (*m*=1, panel **d**). For the first three scenarios a sample size of 100 was used, while in the nested case control sampling a mean of 180 subjects was considered. Data were subject to a random censoring of 15 % (over administrative censoring). Dotted lines represent the reference for CI coverage (nominal 95 %)

Figure 3 reports results on coverage for the random, case-control, stratified and nested case-control sampling. The coverage was very close to the nominal value of 95 %, ranging mostly within a minimum of 94 % and a maximum of 97 %, except for very early times in the random setting.

In the same setting, we also considered a longer follow-up time, t = 50, with around 500 events of type 1 expected in phase I sample (under no censoring), and confirmed the performance of our estimator in a scenario with higher variability, with similar results for the different sampling schemes (data not shown).

### Motivating examples

#### Genotype ascertained on a subset of a clinical trial cohort

A study on childhood ALL evaluated the role of a genetic polymorphism (glutathione S-transferase- *θ*, GST-T1) on treatment failure due to relapse (in different sites), in the presence of a competing event (toxic death). GST-T1 is a common genetic polymorphism in Caucasians, with 13–26 % of individuals displaying a homozygous deletion of the gene (null genotype). Subjects carrying the null variants fail to express the GST-T1 enzyme, that is involved in drug metabolism. Clinical information were available for a cohort of 1999 consecutive patients (mainly European Caucasians, aged between 1 and 17 years, median age: 5 years) newly diagnosed with ALL in the Italian Associazione Italiana di Ematologia Pediatrica centers between September 2000 and July 2006. Biological samples stored at diagnosis were available, but genotype was ascertained only in a subgroup (phase II sample) for an efficient use of specimens [11, 13]. The interest was to evaluate incidence at different relapse sites by GST-T1, that can only be estimated using phase II data. In order to select the subgroup to be genotyped we adopted an optimal strategy that is carefully described in [11, 13]. Briefly, sampling was done after classifying patients into 6 strata according to the event of interest (relapse/no relapse) and to 3 groups, defined by prognostic features in the treatment protocol, that modulate the intensity of treatment, we will call them treatment protocols (Table 1). Strata were not defined based on the competing event death due to toxicity- 58 events - for efficiency reasons given that the event of interest was relapse. Patients were sampled at random without replacement from the 6 strata, with the sampling from each stratum conducted independently (stratified sampling) and with higher probability in the more informative strata according to an optimal design [13]. The full cohort of 1999 patients represents the phase I sample, for which clinical information are available, while genotype is ascertained in the phase II sample only (*n*=601).

**Table 1:** Distribution of phase I (*N*_*s*_) and II (*n*_*s*_) samples in the 6 strata and sampling fractions expressed as percentages in parenthesis for Phase II

	Treatment protocol			
	Standard	Medium	High	
	*n*_*s*_/*N*_*s*_(%)			Total
No relapse	54/487 (11.1)	193/987 (19.6)	109/219 (49.8)	356/1693
Relapse	21/28 (75.0)	147/186 (79.0)	77/92 (83.7)	245/306
Total	75/515	340/1173	186/311	601/1999

**Fig. 4:**
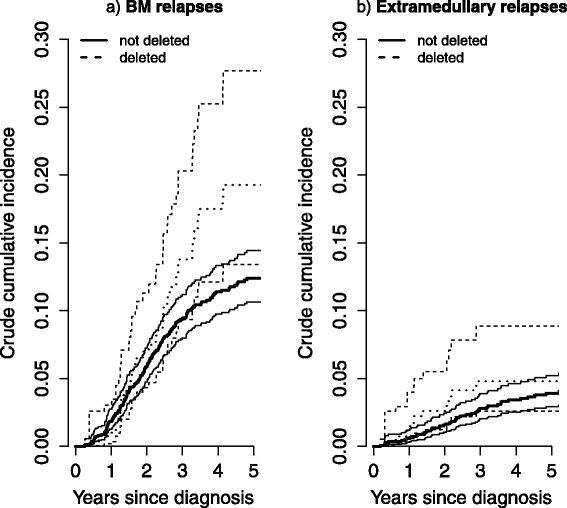
Crude cumulative incidence of relapse in ALL data. Panel **a** reports the crude cumulative incidence estimate of relapse involving the bone marrow in patients with normal (estimate and confidence limits reported with solid lines) and deleted (dashed lines) GST-T1 gene. Panel **b** reports the crude cumulative incidence estimate of relapse in extramedullary sites in patients with normal (solid lines) and deleted (dashed lines) GST-T1 gene

Relapses were classified according to the site, in particular we distinguished relapses involving bone-marrow (BM) from the others (extramedullary). We estimated the crude incidence of BM relapse by GST-T1 deletion using (6) and (10) and found higher relapse incidence for patients with GST-T1 deletion, with 5-year crude incidence of 19.3 *%* (95 % CI: 13.4−27.7 *%*) versus 12.4 *%* (95 % CI: 10.7−14.4 *%* for non deleted patients, Fig. 4 panel a). This was derived accounting for the competing risk of other sites of relapse as well as for death due to toxicity.

We report here the R code used to compute these estimates:library(survey)d.std<-twophase(id=list(~upn,~upn), subset=~!is.na(GST_T),strata=list(NULL,~interaction(rel,elfin)),data=dat)GSTse<-svycr(Surv(time,event>0)~GST_T,etype=“BMrelapse”,d.std,se=TRUE)

The twophase function in the survey package describes the design and produces a survey object [32]. The svycr function, available at [33], performs the estimate of crude incidence by the influence approach and uses 3 variables: time is the time of event, event the censoring indicator (1 if an event of any type is observed and 0 otherwise) and BMrelapse indicates whether a BM relapse is observed or not. Details on the survey package can be found in [7, 32].

The right panel of Fig. 4 represents the incidence of extramedullary relapses by GST-T1 showing that the difference in relapse incidence between GST-T1 deleted and other patients is mainly due to relapse involving the BM, that represents the most relevant type of relapse in childhood ALL. A Cox model adapted for two-phase design [14], when applied to the cause specific hazard of BM relapse, gives an hazard ratio (HR) of 1.53 (95 % CI 0.98–2.37) for GST-T1 deleted patients versus non deleted; after adjusting for relevant factors (treatment protocol, gender, age), the HR dropped to 1.38 (95 % CI 0.90–2.13). For extramedullary relapses the HR was 1.22 (95 % CI 0.60–2.49). Of note, in order to compare patients with and without deletion of the GST-T1 gene, we used a cause-specific model, thus we actually compared the cause-specific hazard of relapse. In fact, a subdistribution model accounting for the two-phase design is not available. This would be useful to compare the actual incidences of relapse in the two groups, however the cause-specific model is still very useful to address the impact of the genotype on relapse by an aetiological point of view.

#### Outcome ascertained on a subset of patients lost to follow-up

In the evaluation of the effectiveness of the global effort to provide antiretroviral therapy (ART) for HIV-infected patients in resource limited settings, the estimate of the number of patients who continue to access care after starting ART is essential. This estimate is hampered, however, by the fact that some patients die shortly after their last visit to clinic - a group of individuals who cannot be considered as “stopping care” nor censored for the event of stopping care. In addition, the number of patients who are starting care is large and a high fraction have unknown outcomes (i.e., are lost to follow-up), generating informative censoring. Given that lost patients could reasonably be not in care, but they could also have changed clinic or be dead, one approach to obtain outcomes estimates has been to identify a numerically small, but random, sample of those who are lost [15], intensively seeking their outcomes, and using them to correct outcomes among the lost.

**Fig. 5:**
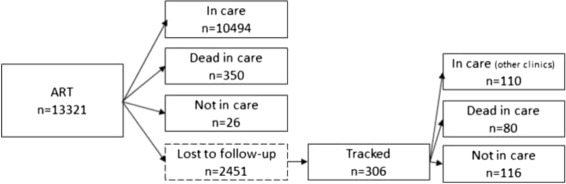
Outcome in the cohort of HIV patients. Outcome of the 13,321 HIV patients who initiated ART treatment

To illustrate, a cohort of 13,321 HIV-infected adult patients, who initiated ART treatment, were followed from ART initiation to either death, disengagement or administrative database closure (see Fig. 5). Among them, 2451 patients were lost to follow-up [35], defined as not being seen at the clinic for at least 90 days (after the last return visit). A tracker went into the community to determine the outcome of a random subsample of 428 among the 2451 lost patients and got information on 306 patients (110 patient were found to be in care in other clinics, 80 died while in care and 116 were found to be not treated/disengaged) [12]. The 10,870 patients no lost to follow-up and the 306 tracked patients can been considered as the second phase sample of the whole cohort, stratified on lost to follow-up.

**Fig. 6:**
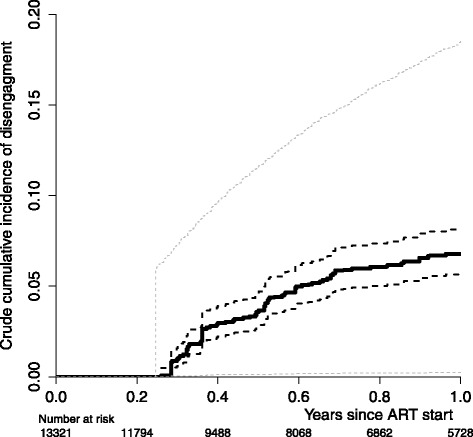
Crude cumulative incidence of disengagement in the HIV data. Crude cumulative incidence of disengagement of the 13,321 HIV patients who initiated ART treatment (black line) with confidence intervals (dashed lines). In the bottom part of the plot the number of patients at risk in time is reported (weighed to represent the whole cohort of 13,321). The dashed grey lines report the crude cumulative incidence of disengagement computed by treating all lost patients as event or censored observation, respectively

We used the methods developed in the Methods section to estimate crude cumulative incidence, where the 306 tracked patients represented the 2451 lost patients by the sampling probability 306/2451, while the other 10,870 had sampling weight one. The crude incidence estimate of disengagement is reported in Fig. 6, the curve starts to rise after 90 days from ART start, that is the earliest possible time of disengagement, by definition. At 1 year, disengagement resulted 6.8 % (CI 95 % 5.7–8.2 %). This was subject to a strong influence of the competing event death in care that resulted 7.7 % (CI 95 % 6.8–8.9 %) at 1 year. A naïve (but less expensive) approach to deal with informative censoring would be to treat all lost patients as events or, contrarily, as censored observations. We plotted the two corresponding curves in Fig. 6, obtaining estimates of crude incidence at 1 year since ART treatment of 18.5 % and 0.2 %, respectively. We can consider that the true incidence will lie between these two estimates (that are however quite far in this context), as in fact it does the estimate we got by tracking a random sample of lost patients and using the proposed estimator.

## Availability of supporting data

The R code to compute the proposed estimate of crude cumulative incidence is available at [33]. The results of simulations presented in the Simulations section and the related code are also available at [33].

## Conclusions

We have derived an estimator for cumulative incidence of events based on the subdistribution hazard accounting for competing risks under a general sampling design from an infinite target population. The estimator shows good performance in simulations under different scenarios and the variance, derived by the influence function of the subdistribution hazard and the Horvitz-Thompson theory, was very close to the empirical variance, therefore we expect it to be very close to the one obtainable by replicate weights (e.g. bootstrap) [7]. Confidence intervals, derived on the log scale, provided good coverage in simulations, but alternative confidence intervals might also be considered such as the complementary log-log transformation [36]. The proposed estimator was used to estimate incidence of relapse by genotype in a cohort of childhood ALL patients, where the genotype was ascertained only on a subsample of the cohort chosen by an optimal sampling approach based on relapse as the event of interest. Interestingly, we can also analyze the incidence of the competing event (toxic death) or of the combined endpoint (relapse or toxic death), but the efficiency could be lower unless the subsampling is adapted to this new endpoint by including a further strata on toxic death in the sampling process. This is particularly important since toxic death is a rare event in this context. We should also remember to avoid random sampling when the event of interest is rare, as discussed in the Simulations section.

In the second case, we dealt with a missing data problem, in which the outcome itself was not available for everybody, since some patients were lost to follow-up. A subsample of lost patients had been tracked to ascertain the outcome, but if this tracking was not possible, a more basic/naïve approach to deal with this informative censoring could have been to identify the variables affecting missingness, post-stratify the sample in homogeneous strata and use the missing probabilities for each strata as sort of sampling weights to adjust the incidence estimate. This approach would make an important assumption of missing at random that might be not appropriate and cannot be tested [7, 15, 21, 37]. However this underlines how the proposed estimator could be applied also in the presence of missing data.

The code to compute this estimate has been developed in R software [31] under the survey package [32] and is available at [33]. The survey package is a flexible package for complex surveys including also two-phase studies. It provides flexible functions to describe the design of the study and to derive sampling fraction accordingly. The package includes functions to estimate survival and to perform a weighted Cox model with standard error properly adjusted for the design and with the possibility to use general weights, as calibrated weights. Our function takes advantage of the facilities of the package (see an example of use in Genotype ascertained on a subset of a clinical trial cohort).

In order to recover the representativeness of the subcohort (phase II) for the entire cohort, we used weights related to the inverse of the probability to be sampled, similarly to the weights of Barlow for case-cohort studies [38]. More general weights can be used, such as calibration weights [7, 39, 40]. The use of calibration weights is advantageous when there is availability of phase I variables that are strongly related to the additional variables ascertained in phase II. This would provide results more representative of phase I data and increase precision. When phase II variables are common genetic polymorphisms, as in our first example, it is unlikely to find any strong relation between phase I and II variables, therefore no big advantage would be expected by calibration.

The estimator can also be extended to a situation where an individual may move among a finite number of states to estimate the Aalen-Johansen probabilities of transition among each state in a multistate framework [41] in the presence of general sampling design.

In order to derive a model-based estimate of incidence (adjusted for possible covariates) two main approaches have been followed in the context of competing risk, the first one based on the cause-specific hazard inspired by Benichou and Gail [16, 42, 43] and the other one based on the subdistribution hazard [19, 44]. The crude cumulative estimator developed by Kovalchik and Pfeiffer [45] for two-phase studies for finite population follows the first approach, and the Cox model for two-phase designs [14] could be used to extend it for infinite population. Under this model we can estimate the effect of a covariate on the cause-specific hazard to address its impact on the event by an aetiological point of view. However it is well known that this does not reflect the impact of the variable on the crude cumulative incidence. The latter effect, even if affected by the incidence of the other competing events, could still be of interest for a public health prospective. Future work will concern the development of a regression model to assess the effect of a covariate on the crude cumulative incidence. The Fine and Gray regression model [19] could be extended to complex sampling by weighting the estimating function of the parameter of interest and working out their influence function.

## A Appendix

### A.1 Derivation of the risk set for $T^{*}_{k}$

The risk set for the usual survival time *T* at *s* is commonly obtained in standard analysis by counting the observed times greater then *s*. It can be also written as:

(12) $$ \hat{Y}_{\cdot}(s)=\hat{P}(T>s^{-})\hat{P}(C>s^{-})={\hat{Y}_{\cdot}(0)} \hat{S}(s^{-})\hat{G}(s^{-})  $$

where $\hat {G}(s)$ is the probability to be free of censoring up to *s* and is estimated considering censored observations as events and viceversa according to (3). This can proved also in the case of a two-phase design by the following: 
(13)

where $\hat {N}_{\cdot }^{c}(t)=\sum _{i=1}^{N} \left [\xi _{i} I(X_{i}\le t,\Delta _{i}=0)/\pi _{i}\right ]$ denotes the number of censoring up to time *t* and $\hat {N}_{\cdot \cdot }(t)=\sum _{i=1}^{n} \hat {N}_{\textit {i}\cdot }(t)$ the total count of events observed up to time *t*.

The derivation of the risk set for $T^{*}_{k}$ at *s* can be obtained by:

(14) $$ {{\begin{aligned} \hat{Y}^{*}_{\cdot k}(s)&=\hat{P}\left(T^{*}_{k}>s^{-}\right)\hat{P}(C>s^{-})={\hat{Y}_{\cdot}(0)} \cdot \left[1-\hat{F}_{k}(s^{-})\right] \hat{G}(s^{-})=\\ &={\hat{Y}_{\cdot}(0)} \cdot\left[\hat{S}(s^{-})+\sum_{l\neq k}\hat{F}_{l}(s^{-})\right]\cdot \hat{G}(s^{-})=\\ &=\hat{Y}_{\cdot}(s)+{\hat{Y}_{\cdot}(0)} \cdot\sum_{l\neq k}\hat{F}_{l}(s^{-})\cdot\hat{G}(s^{-}) \end{aligned}}}  $$

By writing $\hat {F}_{l}(s^{-})$ as empirical cumulative distribution function $\hat {F}_{l}(s^{-})=\frac {1}{\hat {Y}_{\cdot }(0)}\sum _{i=1}^{N} \frac {\xi _{i}}{\pi _{i}}\frac {I(X_{i}\le s^{-};\varepsilon _{i}=l)}{\hat {G}(X_{i}^{-}\wedge s^{-})} $ [25, 46], the risk set becomes:

(15) $$ \begin{aligned} \hat{Y}^{*}_{\cdot k}(s)&={\hat{Y}_{\cdot}(s)}+\sum_{l\neq k}\sum_{i=1}^{N}\frac{\xi_{i}}{\pi_{i}}I(X_{i}\le s^{-};\varepsilon_{i}=l)\frac{\hat{G}(s^{-})}{\hat{G}\left(X_{i}^{-}\wedge s^{-}\right)} \end{aligned}  $$

that can be simplified to

(16) $$ \sum_{i=1}^{N} \frac{\xi_{i}}{\pi_{i}} Y_{i}(s) +\sum_{i=1}^{N} \frac{\xi_{i}}{\pi_{i}} \left[ \sum_{l\neq k} N_{il}(s^{-})\cdot \hat{G}\left(s^{-}|X_{i}^{-}\right)\right]  $$

that is equivalent to (4).

The first summation estimates the usual total number of subjects at risk at *s*, where the condition *X*_*i*_= min(*T*_*i*_,*C*_*i*_)≥*s* is satisfied. This in fact implies $\min (T_{\textit {ki}}^{*},C_{i})\geq s$, i.e. being at risk for *T* implies being also at risk for $T^{*}_{k}$. The second summation estimates the number of subjects who had other events before *s*, satisfying the condition *X*_*i*_= min(*T*_*i*_,*C*_*i*_)<*s*, *Δ*_*i*_=1 and *ε*_*i*_≠*k* which implies $T_{\textit {ki}}^{*}=\infty > s$ and completes the number at risk at *s* for $T^{*}_{k}$. While the first part is exposed to censoring, the contribute of each subject observed to fail of cause *l*≠*k*, $\sum _{l\neq k} N_{\textit {il}}(s^{-})$, would remain equal to 1 up to *∞*, thus ignoring possible censoring, given that *C*_*i*_ is (usually) not observable if *T*_*i*_<*C*_*i*_. A possible way to deal with this inconsistency is to mimic the presence of random censoring acting on the infinite times, by weighting the unitary contributions $\sum _{l\neq k}N_{\textit {il}}(s^{-})$ by the estimate $\hat {G}(s^{-}|X_{i}^{-})$ of $P(C>s^{-}|C>X_{i}^{-})=G(s^{-}|X_{i})$, where *G*(*t*)=*P*(*C*>*t*) is estimated by $\label {eq:G}\hat {G}(t)=\prod _{s\leq t} \left [1- \frac {\sum _{i=1}^{N}\xi _{i} {N_{i}^{c}}(ds)/\pi _{i}}{\sum _{i=1}^{N}\xi _{i} Y_{i}(s)/\pi _{i}}\right ]$, with ${N_{i}^{c}}(s)=I(X_{i}\leq s,\Delta _{i}=0)$. This weight assumes value 1 before *X*_*i*_ and decreases afterword according to the censoring distribution.

Of note, an alternative expression for $\hat {Y}^{*}_{\cdot k}(s)$ derives substituting $\hat {Y}_{\cdot }(0)\hat {G}(s^{-})=\frac {\hat {Y}_{\cdot }(s)}{\hat {S}(s^{-})}$ from (12) in (14):

(17) $$  \hat{Y}^{*}_{\cdot k}(s)={\hat{Y}_{\cdot}(0)}\hat{G}(s^{-}) \cdot\left[1-\hat{F}_{k}(s^{-})\right]={\hat{Y}_{\cdot}(s)} \frac{\left[1-\hat{F}_{k}(s^{-})\right]}{\hat{S}(s^{-})}  $$

This shows as $\hat {Y}_{\cdot }(s)$ is upweighted by a multiplier that gets greater as the action of competing events gets larger, accounting for the fact that subjects that experienced events of type *l*≠*k* will never experience event *k* as first.

### A.2 Proof of equivalence (7)

The equality between the cumulative incidence of the artificial variable *T*^∗^ in (6) and the Aalen-Johansen type estimator (that for the purpose of this proof will be denoted as $\hat {F}^{AJ}_{k}(t)$) holds true if and only if, ∀*t*: 
(18)

which can be proved by induction. Both quantities are step functions, changing value at each occurrence of type *k* events. At the time *t* where the first event of type *k* is observed, $\hat {\Lambda }_{k}(dt)=\hat {\Lambda }^{*}_{k}(dt)$ being from (4) $\hat {Y}_{\cdot }(t)=\hat {Y}_{\cdot k}^{*}(t)$. If this was the first event overall, then $\hat {S}(0)=1$ and Eq. 18 is satisfied, otherwise $\hat {F}^{AJ}_{k}(t)=[\!1-1/\hat {Y}_{\cdot }(0)]\cdot 1/[\!\hat {Y}_{\cdot }(0)-1]=1/\hat {Y}_{\cdot }(0)=1-[\!1-1/\hat {Y}_{\cdot }(0)]=\hat {F}_{k}(t)$.

Now, assuming that (18) holds true for a given *t*^−^, this implies $\hat {F}^{AJ}_{k}(t)=\hat {F}_{k}(t)$ if and only if, from (18):

$$\begin{aligned} \hat{F}^{AJ}_{k}(t^{-})+\hat{S}(t^{-})\hat{\Lambda}_{k}(dt)& = 1-(1-\hat{F}_{k}(t^{-}))\left(1-\hat{\Lambda}^{*}_{k}(dt) \right) \end{aligned} $$

and using the equality at *t*^−^:

$$\begin{aligned} \hat{F}_{k}(t^{-})+\hat{S}(t^{-})\hat{\Lambda}_{k}(dt) &=\hat{F}_{k}(t^{-})+\left[1-\hat{F}_{k}(t^{-})\right]\hat{\Lambda}^{*}_{k}(dt)\\ \hat{S}(t^{-})\hat{\Lambda}_{k}(dt) &= \left[1-\hat{F}_{k}(t^{-})\right]\hat{\Lambda}^{*}_{k}(dt)\\ \hat{\Lambda}_{k}(dt)& = \frac{1-\hat{F}_{k}(t^{-})}{\hat{S}(t^{-})}\hat{\Lambda}^{*}_{k}(dt)\\ \frac{\hat{N}_{k}(dt)}{\hat{Y}_{\cdot}(t)} &=\frac{1-\hat{F}_{k}(t^{-})}{\hat{S}(t^{-})}\frac{\hat{N}_{k}(dt)}{\hat{Y}^{*}_{\cdot k}(t)} \\ \hat{Y}^{*}_{\cdot k}(t) & = \hat{Y}_{\cdot}(t) \cdot \frac{1-\hat{F}_{k}(t^{-})}{\hat{S}(t^{-})} \end{aligned} $$

That is proved by (17).

### A.3 Weak convergence of $\hat {\Lambda }_{k}^{*}(t)$

Lin showed that the normalised Horvitz-Thompson estimators of the number of events $\sqrt {N}\left [\hat {N}_{\cdot }(t)-N_{\cdot }(t)\right ]$, number at risk $\sqrt {N}\left [\hat {Y}_{\cdot }(t)-Y_{\cdot }(t)\right ]$, cumulative hazard $\sqrt {N}[\hat {\Lambda }(t)-\Lambda (t)]$ and survival function $\sqrt {N}\left [\hat {S}(t)-S(t)\right ]$ (and analogously $\sqrt {N}\left [\hat {G}(t)-G(t)\right ]$) are asymptotically multivariate zero-mean normal [14]. Firstly, we concentrate on the normalised Horvitz-Thompson estimators of the modified at risk process: $\sqrt {N} \left [\hat {Y}^{*}_{\cdot k}(t)-Y^{*}_{\cdot k}(t)\right ]=\sqrt {N}\sum _{i=1}^{N}\frac {\xi _{i}-\pi _{i}}{\pi _{i}}Y^{*}_{\cdot k}(t) =\sqrt {N}\sum _{i=1}^{N} \frac {\xi _{i}-\pi _{i}}{\pi _{i}}Y_{\cdot k}(t)+\sqrt {N}\sum _{i=1}^{N}\frac {\xi _{i}-\pi _{i}}{\pi _{i}}\sum _{l\neq k}N_{i l}(t^{-}) \hat {G}(t^{-}|X_{i})$. The first term represents the estimator of the number at risk, that Lin showed to be asymptotically multivariate zero-mean normal [14]. We concentrate now on the second term:

$${\fontsize{8.8pt}{9.6pt}{\begin{aligned} &\sqrt{N}\sum_{i=1}^{N} \left[ \frac{\xi_{i}}{\pi_{i}} \cdot \sum_{l\neq k} N_{i l}(t^{-}) \hat{G}(t^{-}|X_{i}) -\sum_{l\neq k} N_{i l}(t^{-}) \hat{G}(t^{-}|X_{i}) \right]= \\&=\sqrt{N}\sum_{i=1}^{N} \left[\hat{G}(t^{-}|X_{i})\left\lbrace \frac{\xi_{i}}{\pi_{i}} \sum_{l\neq k} N_{i l}(t^{-}) -\sum_{l\neq k} N_{il}(t^{-})\right\rbrace\right]=\\ &=\sqrt{N}\left[\hat{G}(t^{-}|X_{i})\left\lbrace \sum_{l\neq k} \hat{N}_{\cdot l}(t^{-}) - \sum_{l\neq k} \sum_{i=1}^{N} N_{i l}(t^{-})\right\rbrace\right]= \\ &=\sqrt{N}\int_{0}^{t^{-}}\hat{G}(s^{-}|X_{i})\left\lbrace \sum_{l\neq k} \hat{N}_{\cdot l}(ds{-}) - \sum_{l\neq k} \sum_{i=1}^{N} N_{i l}(ds^{-})\right\rbrace=\\ &=\sqrt{N}\int_{0}^{t^{-}} G(t^{-}|X_{i})\left\lbrace \sum_{l\neq k} \hat{N}_{\cdot l}(ds{-}) - \sum_{l\neq k} \sum_{i=1}^{N} N_{i l}(ds^{-})\right\rbrace+\\ &\qquad+o_{p}(1) \end{aligned}}} $$

that using Lemma 1 in [14] also converges to a zero-mean Gaussian process.

We want to prove that also $\sqrt {N}\left [\hat {\Lambda }^{*}_{k}(t)-\Lambda ^{*}_{k}(t)\right ]=\sqrt {N}\left [\tilde {\Lambda }^{*}_{k}(t)-\Lambda ^{*}_{k}(t)\right ] +\sqrt {N}\left [\hat {\Lambda }^{*}_{k}(t)-\tilde {\Lambda }^{*}_{k}(t)\right ]$ converges to a zero-mean normal, where $\tilde {\Lambda }^{*}_{k}(t)$ represents the crude cumulative incidence estimator that we would have obtained if complete information (*X*_*i*_,*Δ*_*i*_*ε*_*i*_,*Z*_*i*_) was known for all the subjects in phase I sample (*i*=1…*N*) [18]. Fine and Gray proved that the first term converges weakly to a zero-mean Gaussian process [19].

The second term results:

$${\fontsize{8.6pt}{9.6pt}{\begin{aligned} \sqrt{N}&\left[\hat{\Lambda}^{*}_{k}(t)-\tilde{\Lambda}^{*}_{k}(t)\right]= \sqrt{N}\left[\int_{0}^{t}\frac{\hat{N}_{k}(ds)}{\hat{Y}^{*}_{\cdot k}(s)}-{\int_{0}^{t}}\frac{N_{k}(ds)}{Y^{*}_{\cdot k}(s)}\right]=\\ & =\sqrt{N}\left[\int_{0}^{t}\frac{\hat{N}_{k}(ds)}{\hat{Y}^{*}_{\cdot k}(s)}-{\int_{0}^{t}}\frac{N_{k}(ds)}{Y^{*}_{\cdot k}(s)} +{\int_{0}^{t}}\frac{{N}_{k}(ds)}{\hat{Y}^{*}_{\cdot k}(s)} -{\int_{0}^{t}}\frac{{N}_{k}(ds)}{\hat{Y}^{*}_{\cdot k}(s)}\right]=\\ & =\sqrt{N}\left[\int_{0}^{t} \frac{\hat{N}_{k}(ds)-N_{k}(ds)}{\hat{Y}^{*}_{\cdot k}(s)} - {\int_{0}^{t}}\frac{N_{k}(ds)\left[\hat{Y}^{*}_{\cdot k}(s)-Y^{*}_{\cdot k}(s)\right]}{\hat{Y}^{*}_{\cdot k}(s)Y^{*}_{\cdot k}(s)}\right]. \end{aligned}}} $$

It then follows that also $\sqrt {N}\left [\hat {\Lambda }^{*}_{k}(t)-\tilde {\Lambda }^{*}_{k}(t)\right ]$ converges weakly to a zero-mean Gaussian process.

### A.4 Influence function for $\hat {\Lambda }_{k}^{*}(t)$

The estimator $\hat {\Lambda }_{k}^{*}(t)$ can be expressed as a differentiable function *g* of the estimated total number of events of type *k* and total number at risk for *T*^∗^ up to *t*:

(19) $$ \begin{aligned} \hat{\Lambda}^{*}_{k}(t)=g(\hat{N}_{\cdot k}(dt),\hat{Y}^{*}_{\cdot k}(t))&={\int_{0}^{t}}\frac{ \hat{N}_{\cdot k}(ds)}{\hat{Y}_{\cdot k}^{*}(s)}=\\ &={\int_{0}^{t}}\frac{\sum_{i=1}^{N} \xi_{i} \cdot N_{ik}(ds)w_{i}}{\sum_{i=1}^{N} \xi_{i} \cdot Y_{ik}^{*}(s)w_{i}} \end{aligned}  $$

where *w*_*i*_=1/*π*_*i*_ and *ξ*_*i*_ indicates whether subject *i* is withdrawn in the phase II sample.

The difference between the true and estimated cumulative hazard can be expressed as a sum of influence functions: $\hat {\Lambda }_{k}^{*}(t)-\Lambda ^{*}_{k}(t)=\sum _{i=1}^{N} z_{\textit {ik}}^{*}+o(1/\sqrt {N})$, where $z_{\textit {ik}}^{*}$ is the influence function of the *i*^*th*^ subject. Demnati and Rao [27] proved that we can express the influence function of subject *i* as $z_{\textit {ik}}^{*}(t)=\frac {\partial g \left (\hat {N}_{\cdot k}(dt),\hat {Y}^{*}_{\cdot k}(t)\right)}{\partial w_{i}}$. The influence function of $\hat {\Lambda }^{*}_{k}(t)$ of the *i*^*th*^ subject can thus be derived as:

(20) $$ z_{ik}^{*}(t)={\int_{0}^{t}} \frac{N_{ik}(ds)\hat{Y}^{*}_{\cdot k}(s)-\frac{\partial \hat{Y}^{*}_{\cdot k}(s)}{\partial w_{i}} \hat{N}_{\cdot k}(ds)}{\hat{Y}^{*}_{\cdot k}(s)^{2}}  $$

Being $\hat {Y}^{*}_{\cdot k}(s)=\sum _{i=1}^{N} \xi _{i} w_{i}\cdot \left [ Y_{i}(s)+\frac {I(X_{i}\le s^{-};\varepsilon _{i}\neq k)\hat {G}(s^{-})}{\hat {G}(X_{i}^{-}\wedge s^{-})} \right ]$ and being , the derivative of $\hat {Y}^{*}_{\cdot k}(s)$ with respect to *w*_*i*_ results:

(21) $$ \begin{aligned} \frac{\partial \hat{Y}^{*}_{\cdot k}(s)}{\partial w_{i}}&= \frac{\partial \left\{ \xi_{i} w_{i}\cdot\left[ Y_{i}(s)+\frac{I(X_{i}\le s^{-};\varepsilon_{i}\neq k)\hat{G}(s^{-})}{\hat{G}\left(X_{i}^{-}\wedge s^{-}\right)} \right]+ \underset{j\neq i}{\sum} \xi_{j} w_{j}\cdot\left[Y_{j}(s)+\frac{I(X_{j}\le s^{-};\varepsilon_{j}\neq k)\hat{G}(s^{-})}{\hat{G}\left(X_{j}^{-}\wedge s^{-}\right)}\right]\right\}}{\partial w_{i}}=\\ & =\xi_{i}Y_{i}(s)+\xi_{i}\frac{\partial \left[w_{i} \frac{I(X_{i}\le s^{-};\varepsilon_{i}\neq k) \hat{G}(s^{-})}{\hat{G}\left(X_{i}^{-}\wedge s^{-}\right)}\right]}{\partial w_{i}} +\frac{\partial \left[\underset{j\neq i}\sum \frac{\xi_{j} w_{j} I(X_{j}\le s^{-};\varepsilon_{j}\neq k)\hat{G}(s^{-})}{\hat{G}\left(X_{j}^{-}\wedge s^{-}\right)}\right]}{\partial w_{i}}=\\ &=\xi_{i}Y_{i}(s)+\xi_{i}\frac{I(X_{i}\le s^{-};\varepsilon_{i}\neq k)\hat{G}(s^{-})}{\hat{G}\left(X_{i}^{-}\wedge s^{-}\right)}+ \sum_{j=1}^{N}\xi_{j} w_{j} I(X_{j}\le s^{-};\varepsilon_{j}\neq k)\frac{\partial}{\partial w_{i}} \left[\frac{\hat{G}(s^{-})}{\hat{G}\left(X_{j}^{-}\wedge s^{-}\right)} \right]. \end{aligned}  $$

Please note that the last addendum accounts for the fact that $\hat {G}(t)$ is estimated using information of subject *i*. For *v*≤*u*:

(22) $$ \begin{aligned} \frac{\partial}{\partial w_{i}}\left[ \frac{\hat{G}(u)}{\hat{G}(v)}\right]=\,&\frac{\hat{G}(u){\int_{0}^{u}} \frac{d{N_{i}^{c}}(s)-\hat{\lambda}^{c}(s)Y_{i}(s)}{\hat{Y}_{\cdot}(s)}\hat{G}(v)-\hat{G}(v){\int_{0}^{v}} \frac{d{N_{i}^{c}}(s)-\hat{\lambda}^{c}(s)Y_{i}(s)}{\hat{Y}_{\cdot}(s)}\hat{G}(u)}{\hat{G}(v)^{2}}=\\ =\,&\frac{\hat{G}(u)\hat{G}(v)\int_{v^{+}}^{u} \frac{d{N_{i}^{c}}(s)-\hat{\lambda}^{c}(s)Y_{i}(s)}{\hat{Y}_{\cdot}(s)}}{\hat{G}(v)^{2}} =\frac{\hat{G}(u)\int_{v^{+}}^{u} \frac{d{N_{i}^{c}}(s)-\hat{\lambda}^{c}(s)Y_{i}(s)}{\hat{Y}_{\cdot}(s)}}{\hat{G}(v)} \end{aligned}  $$

where the superscript *c* indicates the quantities related to the censoring process, i.e. ${N_{i}^{c}}(u)=I(X_{i}\leq u,\Delta _{i}=0)$ is the indicator of censoring for subject *i* up to time *u* and $\hat {\lambda }^{c}(u)=\hat {N}_{\cdot }^{c}(du)/\hat {Y}_{\cdot }(u)$ the instantaneous hazard of censoring. The derivative of $\hat {Y}^{*}_{\cdot k}(u)$ becomes:

(23) $$ \begin{aligned} & \frac{\partial \hat{Y}^{*}_{\cdot k}(s)}{\partial w_{i}}=\\&Y_{i}(s)+ I(X_{i}\le s^{-};\varepsilon_{i}\neq k) \cdot\frac{\hat{G}(s^{-})}{\hat{G}\left(X_{i}^{-}\wedge s^{-}\right)} +\sum_{j=1}^{N}\xi_{j} w_{j} I(X_{j}\le s^{-};\varepsilon_{j}\neq k)\frac{\hat{G}(s^{-})}{\hat{G}\left(X_{j}^{-}\wedge s^{-}\right)}\left[\int_{X_{j}}^{s^{-}} \frac{{N_{i}^{c}}(du)-\hat{\lambda}^{c}(u)Y_{i}(u)}{\hat{Y}_{\cdot} (u)}\right] \end{aligned}  $$

Thus, the influence function of $\hat {\Lambda }^{*}_{k}(t)$ of the *i*^*th*^ subject results: 
(24)

where *w*_*j*_=1/*π*_*j*_. By defining ${M_{i}^{c}}(s,t)={\int _{s}^{t}} \frac {{N_{i}^{c}}(du)-\hat {\lambda }^{c}(u)Y_{i}(u)}{\hat {Y}_{\cdot } (u)}$ as the influence function of subject *i* on censoring, $v_{j}=\frac {\xi _{j}}{\pi _{j}} \sum _{l \neq k}N_{\textit {jl}}(s^{-})\hat {G}(s^{-}|X_{j}^{-})$, and $M_{\textit {ik}}(s)=\left [N_{\textit {ik}}(s)-\hat {\Lambda }_{k}^{*}(s)Y^{*}_{\textit {ik}}(s)\right ]/\hat {Y}^{*}_{\cdot k}(s)$.

Thus it can be reduced as: 


## Abbreviations

ALL: Acute lymphoblastic leukemia
ART: Antiretroviral therapy
BM: Bone-marrow
CI: Confidence interval
GST-T1: Glutathione S-transferase- *𝜃*
HR: Hazard ratio
